# Child neurology services for children with epilepsy in Finland

**DOI:** 10.1002/epi4.12436

**Published:** 2020-10-12

**Authors:** Matti Sillanpää, Maiju M. Saarinen, Tuire Lähdesmäki

**Affiliations:** ^1^ Department of General Practice University of Turku Turku Finland; ^2^ Department of Child Neurology Turku University Hospital Turku Finland

**Keywords:** child health care, child neurologist density, child neurologist training, health services, pediatric neurology

## Abstract

**Objective:**

The aim of the study was to describe healthcare organization, training of and needs for child neurologists, patient accessibility to services, and treatment paths of children with epilepsy in Finland.

**Methods:**

Data were collected from all geographic healthcare areas over Finland on training capacity in child neurology, number and density of child neurologists, and availability and accessibility of child neurological services. Data sources included the National Physician Register, Central Register of Healthcare Professionals of National Supervisory Authority for Welfare and Health, and phone and email inquiries to the heads of public healthcare units.

**Results:**

The overall density of child neurologists in Finland was 11.9/100 000 children aged 0‐15 years or 8402 children per child neurologist (in 2018). There is a remarkable geographic variation, from 7.1 in northern Finland to 15.6 in the metropolitan area. However, waiting times for the treatment are virtually the same all over the country. According to the Finnish current practice recommendation from the year 2013 and again 2020, children with any first nonfebrile or complicated febrile epileptic seizure are invariably admitted to hospital for evaluation. Children with simple febrile seizures are recommended to be treated as outpatients by general practitioners or by experienced pediatricians.

**Significance:**

Child neurology services are today well provided and organized in Finland. While there is geographic variation in the number of child neurologists, the accessibility is virtually the same all over the country. A gap between the numbers of specialists at near‐to‐retire age and those in training is a challenge.

**Key points**

- Child neurology is a recognized full‐time specialty in Finland.
- Training of child neurologists largely meets the needs.
- Access to the services is reasonably good all over the country.
- Virtually all children with epilepsy are treated by child neurologist.
- Training capacity with regard to the needs will be a challenge in the future.

## 1 INTRODUCTION

As an integrated area of medicine, child neurology has its roots in the early 1800s but, as a distinct discipline, it can be characterized after World War II, in the 1950s and 1960s.^1^ During the past five decades, little has been reported about the resources of the discipline, and few previous studies explore the frequency and training of child neurologists with regard to the needs in the population.^2^, ^3^, ^4^, ^5^, ^6^

The start of the modern Finnish child neurology, including child epileptology, dates back to the 1960s. In 1967, the formal training program in child neurology (CN) began, the Finnish Child Neurology Association was established, and the first specialists were authorized as clinical child neurologists. Recent discussions in the literature concerning workforce and financial resources for and the future of child neurology induced us to describe the current status of the treatment for children with new‐onset epileptic seizures in Finland. We also aimed to compare the availability, accessibility, and variations in the geographic distribution of the child neurology specialized services in the country.

## 2 MAIN FEATURES OF THE FINNISH CHILD HEALTHCARE SYSTEM

Table 1 shows the organization structure of the CN services in Finland. The establishment of public well‐baby clinics since the middle of the 1940s, extension of the clinics all over the country, and the unbroken chain of services from maternity clinics to well‐baby clinics and further to school health care provide preventive services that well cover infants, children, and adolescents. All the services are part of the countrywide network of public health centers, which deliver free‐of‐charge medical primary care services for patients of all ages and particularly follow‐up of health and development and preventive measures for children. In addition to general practice services, some of the largest cities also offer additional child neurological specialist services.

**TABLE 1:** Organization of the child neurological (CN) health care in 2019 in Finland

Level	Coverage	CN specialty services
Public primary care		
Well‐baby clinics	Countrywide network	Larger urban communities
Health centers	Countrywide network	Larger urban communities
Private clinics	Larger urban communities	Larger urban communities
Secondary care		
Regional hospitals	Southern Finland	One regional hospital
Tertiary care		
Central hospital child neurological wards/beds	Countrywide network	Countrywide network
University hospital child neurological departments	Countrywide network	Countrywide network
Health care for intellectually disabled		
Home care	Countrywide network	Most special care districts
Institutional care	Countrywide network	Most special care districts

The national health system legislation is the base of the Finnish health care. A parallel system of private facilities is available. It is run in most cases part time and beyond office hours by child neurologists employed at public hospitals and less often by full‐time private child neurologists. The private services are partly refunded for the patients by virtue of the social insurance legislation or fully refunded by a voluntary private health insurance, if any. The private office services are favored by the patients and families, who want to use a designed physician's services, who want to have a shortest possible waiting time, and who, in many cases, have a private health insurance. Another reason is the off‐hours availability of the private services as, in Finland, a remarkable percentage of mothers of children are at paid daily work (74% of mothers and 90% of fathers with minor children in 2016, https://www.stat.fi/ajk/julkistamiskalenteri/kuvailusivu_fi.html?ID=19722 ), but are free to seek medical advice after the workday. There are no private hospitals for child neurological patients.

**FIGURE 1:**
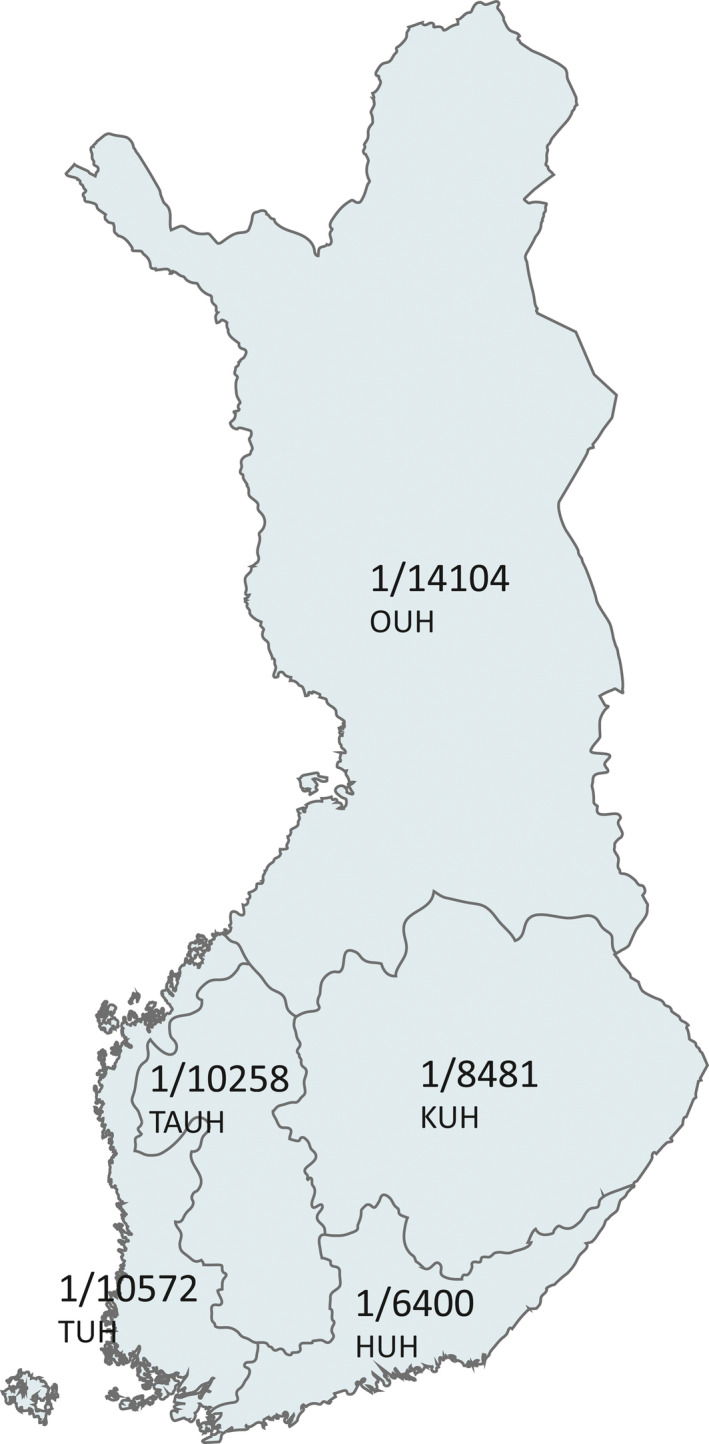
Five specific catchment areas and number of children at age 0‐15 years per trained child neurologist offices in each catchment area in 2018 in Finland. HUH, Helsinki University Hospital; KUH, Kuopio University Hospital; OUH, Oulu University Hospital; TUH, Turku University Hospital; TAUH, Tampere University Hospital

Finland is geographically divided into 20 tertiary care regions. Every regional hospital (central hospital) has the mandate of the designed population in the area, which means that their catchment areas are not overlapping. (Table 2). While a teaching duty belongs to every Finnish public healthcare unit, five of the 20 central hospitals have an extended teaching and research status as university hospitals (Figure 1). No hospitals or institutions exist anymore for epilepsy care only.

**TABLE 2:** Population statistics and number of trained child neurologists (CNs) by hospital catchment areas in Finland at the end of 2018

Specific catchment area	Catchment area	Total population	Child population (<16 y)	Proportion children	CNs	CNs per 100 000 total population	CNs per 100 000 child population
		n	n	%	n		
Helsinki University Hospital Area		2 173 797	371 223	17.1	58	2.67	15.62
	Helsinki and Uusimaa (HUS)	1 667 203	294 866	17.7	50	3.00	16.96
	Kymenlaakso (KYM)	166 623	24 353	14.6	2	1.20	8.21
	South Karelia (EK)	128 756	18 673	14.5	2	1.55	10.71
	Päijänne‐Tavastia (PH)	211 215	33 331	15.8	4	1.89	12.00
Turku University Hospital Area		899 575	148 010	16.5	14	1.56	9.46
	Southwest Finland (VS) and Åland Islands (Å)	511 267	82 184	16.1	10	1.96	12.17
	Satakunta (S)	218,624	34,582	15.8	3	1.37	8.68
	Vaasa (V)	169,684	31,244	18.4	1	0.59	3.20
Tampere University Hospital Area		900 724	153 869	17.1	15	1.67	9.75
	Pirkanmaa (P)	535 044	90 442	16.9	8	1.50	8.85
	Tavastia Proper (KH)	171 364	28 615	16.7	3	1.75	10.48
	Southern Ostrobothnia (EP)	194 316	34 812	17.9	4	2.06	11.49
Kuopio University Hospital Area		805 133	127 211	15.8	15	1.86	11.79
	Southern Savonia (ES)	100 226	14 198	14.2	1	1.00	7.04
	Eastern Savonia (IS)	41,060	5,349	13.0	0	−	−
	Central Finland (KS)	252,676	43,914	17.4	1	0.40	2.28
	North Karelia (PK)	165 569	24 875	15.0	3	1.81	12.06
	Northern Savonia (PS)	245 602	38 875	15.8	10	4.07	25.72
Oulu University Hospital Area		738 690	141 043	19.1	10	1.35	7.09
	Central Ostrobothnia (KP)	77 689	15 544	20.0	0	−	‐‐
	Northern Ostrobothnia (PP)	409 418	85 748	20.9	7	1.71	8.16
	Kainuu (KAI)	73 061	11 041	15.1	1	1.37	9.06
	Southwest Lapland (LP)	61 172	10 338	16.9	1	1.63	9.67
	Lapland (L)	117 350	18 372	15.7	1	0.85	5.44
Total		5 517 919	941 356	17.1	112	2.03	11.90

## 3 CHILD NEUROLOGIST TRAINING

To meet the increasing needs for CN education, both the subspecialty of child neurology as part of neurology and the child neurological association were founded in 1967. In 1978, a recognized full‐time CN specialty was officially established. In Finland, the educational program covers both acute child neurology and neurodevelopmental disability specialties. Specialist examination training is under the supervision of the five medical faculties. The length of the CN training is six years. The requirements for the CN specialist training follow the European Union recommendations. The register of trained and board‐certified child neurologists is maintained by Valvira, a national agency operating under the Ministry of Social Affairs and Health.

The 6‐year specialty training consists of (a) 9‐month service in public primary health care; (b) minimum 12 months as resident in the department of pediatrics; (c) minimum 3 months as resident in the department of neurology; and (d) 36‐48 months as resident in the department of child neurology. Up to 12 of the 48 months can be substituted for by service in the departments of medical neurospecialties, mainly general pediatrics. All the service periods are taken in a university hospital or other hospital accepted by the university for teaching purposes. The minimum uninterrupted period in one CN teaching unit is 6 months. To get authorization to practice as CN specialist, the trainee must have passed six‐year CN training, performed accepted theoretical courses for 60 hours, acquired 30 credits in management training, and passed the national written examination.

**FIGURE 2:**
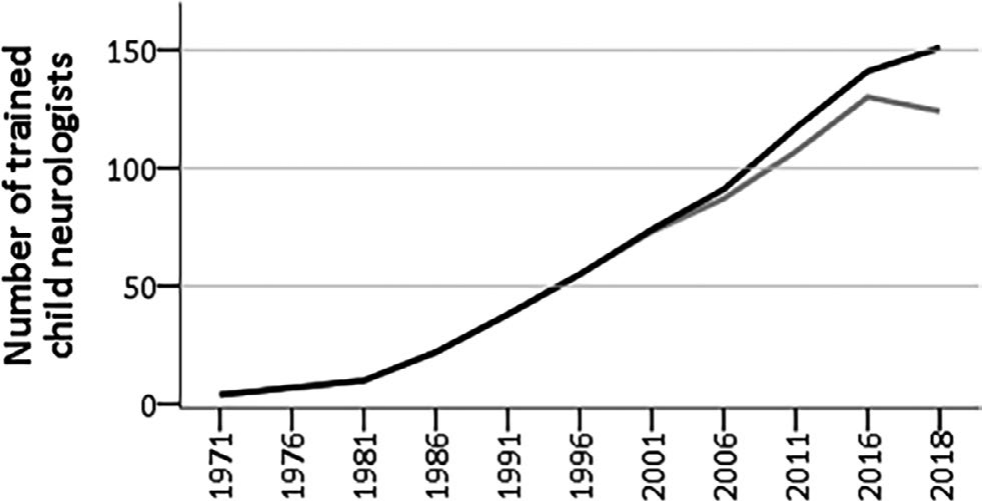
Cumulative increase in number of child neurologists 1967 through 2018 in Finland. Black line = total; gray line = at working age

The total number of trained child neurologists remained relatively low until the 1980s, but then started steeply to increase (Figure 2). In the middle of the 1990s, the density of 1 to 20 000 children less than 16 years was obtained. At the end of 2018, the average density was 112 to 941 376 or 1 to 12 069 8405 children.

A contributing factor to the increasing number of child neurologist specialists has been an increase in training positions. During the years 2014‐2018, the specialist training was completed by 18 trainees. Assuming the same number of new specialists in 2020‐2024 and considering the age of retirement of 20 specialists during the same time interval, there will be a calculated shortage of two specialists. In the beginning of 2019, there are 11 positions for CN residency in the country, mainly at the five university hospitals.

The CN trainees do not incur any financial liabilities for education. During the training period, their income mainly consists of hospital baseline salaries and various bonuses, such as those of off‐hours duties. They may also get supplemental revenues from private office fees. Interest in CN training is continuing to be good in Finland, and no problems in recruitment have appeared.

Compared with other countries, the Finnish CN training program perhaps puts somewhat more emphasis on neurology than pediatrics. In practice, the child neurological and pediatrics units are, however, both physically nearby each other and functionally and administratively in a close collaboration in terms of joint ward rounds, clinical, X‐ray and scientific meetings, case conferences, and similar daily and weekly functions. In most hospitals, child neurologists and pediatricians also share the off‐hours duties. These arrangements also serve the specialist training purposes.

## 4 ORGANIZATION AND FUNCTION OF CHILD NEUROLOGICAL SERVICES

Table 2 presents the number of trained and board‐certified CNs employed in different parts of Finland with regard to the child population of less than 16 years at the end of 2018. The size of the total population and the child population in particular greatly varied in the different catchment areas. Even 1½‐fold regional differences were found in the ratios of number of children per one child neurologist, with the lowest ratios being in eastern Finland and the highest ones in northern Finland (Figure 1).

Except for the Åland Islands, covered by the Turku University Hospital services, there are CN services in all the 20 central hospitals with their catchment areas covering the whole country. Five of the central hospitals have a university teaching hospital status.

Until the end of 2018, altogether 159 physicians were trained and board‐certified as specialists in the recognized main specialty of child neurology in Finland. In the course of years, 39 (25%) had age‐retired, 7 (4%) deceased, and 1 (1%) moved abroad, with the remainder 112 (70%) in the workforce. There was a broad female majority (86%). The placement of offices mainly determines where the child neurologists practice. The vast majority (80%) of the 112 child neurologists were working, all full‐time, within a hospital‐based, mostly academic setting in close proximity to allied health professionals and diagnostic equipment. Ten percent practiced in municipality‐based settings, 5% practiced in civil service departments, and the remaining 5% were at other work including full‐time private office (n = 2) and scientific research or similar activities (n = 3).^7^, ^8^

In the university hospitals, there are designed epilepsy nurses, but in other tertiary care hospitals, the tasks of an epilepsy nurse are shared by two or more pediatric nurses. There is no formal training program for epilepsy nurses, but they have a workplace training and a standing position in the treatment groups. Most hospitals arrange continuing training for epilepsy nurses. A practice is to have a designed nurse for every epilepsy patient during hospitalization and follow‐up. Epilepsy nurse has no role in diagnosing epilepsy, but he or she acts as contact person between the family and hospital, and guides for social benefits, third sector organizations, and taking care of other practicalities.

### 4.1 Availability and accessibility

At the end of the year 2018, in Finland, there were 8405 children per one child neurologist employed in a public office presenting 11.9 child neurologists per 100 000 children under 16 years or 12.7/100 000 children under 15 years (Table 2). Calculated for the total population, the density of trained child neurologists in the working power was 2.03 per 100 000.

With reference to accessibility, geographic distances are no great problem in the southern parts of the country, where there is dense population, effective public communications network, and relatively short geographic distances. Patient transports in general and emergency cases in particular in sparsely populated areas, including the archipelago and Lapland, are organized by surface ambulance transports or by helicopter flights. Usage of public communications, taxi, or own car is partly reimbursed by the national health system. Accessibility to diagnostic investigations and initiation of treatment is within few days all over the country, even in sparsely populated areas. Accessibility to diagnostic investigations and initiation of treatment is within few days all over the country, even in sparsely populated areas. An email query directed in the five university hospital areas, including 1‐3 tertiary care centers each, showed no delay in elective care in six centers, a delay of less than five percent of all appointments in two, and a delay of 20% in one center. In practice, this means approximately one to two weeks on the waiting list.

### 4.2 Identification and validation of cases

To standardize the clinical CN diagnose making, a guide of the best practice recommendations is published and regularly updated. In addition, the Child Neurological Association, Finnish Epilepsy Association, and Finnish Epilepsy Society arrange multiannual meetings, all with a good attendance.

A child who has a first epileptic seizure is in practically all cases admitted to hospital for the evaluation of a trained child neurologist. There is only one exception of the rule, namely simple febrile seizures that are, according to the current recommendations, treated in ambulatory care by trained pediatricians or general practitioners. A conventional EEG with sleep and provocation tests is carried out in every case with complicated febrile seizure or any suspected or ascertained nonfebrile seizure, not least for differential diagnostic purposes, and EEG exploration may be complemented on clinical grounds with video‐EEG investigations. A magnetic resonance imaging (MRI) investigation is a rule in case of suspected focal origin of seizures. In selected cases, functional MRI, positron emission tomography study, and metabolic and genetic testing are performed to uncover the etiology. The diagnosis of seizure disorder and possible comorbidities is made by the treating doctor alone or in collaboration with the pediatric epilepsy team, if needed. Antiepileptic drug therapy is administered only when a consensus has been achieved with the family and, if relevant, the patient.

### 4.3 Clinical data documentation

Child neurologist is in charge of the childhood‐onset epilepsy diagnoses. In Finland, the treating doctor personally documents the diagnosis codes in the patient records and remains in legal charge of the correctness of the data. If the diagnosis is missing, the electrical records system does not approve the data transfer to the national all‐records data system but returns the data to the treating doctor for revision. The diagnosis/diagnoses are also necessary for the healthcare unit to get the care costs paid by the national healthcare system. All hospital patient records on inpatients are collected since 1967 and outpatient visits since 1998 in the general nationwide patient data records register maintained by the Institute of Health and Welfare.^9^ In addition to the general national register, a national epilepsy records register is under preparation. As of 2020, it works in three of the five university hospital regions: Helsinki, Kuopio, and Turku.

## 5 THIRD SECTOR ORGANIZATION

Children with epilepsy and their families may also get social support from the nationwide third sector organization and its regional subassociations all over the country. During the history of more than one hundred years of organized activities for people with epilepsy, the present Epilepsy Association of Finland is an influential actor and advocate of its members, not least in social affairs. It has a close collaboration with the public health care.

## 6 DISCUSSION AND CONCLUSIONS

The modern Finnish child neurology has a 50‐year history. It was initially based mainly on general pediatrics and has never, during its existence, shown any tendencies to be separated from pediatrics. While adult neurology was, in the very beginning, an alternative for pediatrics as a background specialty, all child neurologists had a preceding training in pediatrics. Since 1978, when it became possible to enter the CN training program without preceding pediatric specialty, pediatrics has still a firm position at daily clinical work and specialist training of child neurologists. This fact is of importance from the view of collaboration, straight consultations, and flexible decision making between child neurologists and pediatricians.

The CN density in Finland, 11.9/100 000 children under 16 years, is far more than the recommendations (1/100 000) suggested at the beginning of the 1970s^2^, when the CN’s main role was to ascertain the clinical diagnosis^3^. Today, the work field is much broader and more complex than previously^10^ and, consequently, the needs for CN services are higher. Overall, few data exist on the density of full‐time child neurologists and needs of CN services (Table 3). We made massive efforts to identify similar reports from other countries than those included in Table 3, but we did not find any. Undoubtedly, the national data on the number of child neurologists are based on somewhat different grounds, as are also the requirements for the specialists. Compared with other countries, the density of child neurologists seems to be definitely highest in Finland. Unfortunately, from most countries, the useful and updated data were not available to us.

**TABLE 3:** Density of child neurologists per 100 000 children (0‐14 years) and per 100 000 total population

Country and year	No. of child neurologists	Members in CN specialist association	Child population 0‐14 y	Total population	Density/child population	Density/total population	Reference
Canada^a^ 2014	135		5 607 345	33 476 685	2.41	0.40	Doja et al (2016)^5^
USA 2015	1625		60 999 735	320 742 673	2.66	0.51	Kang et al (2016)^12^
Estonia 2018	9		215 636	1 322 920	4.17	0.68	Statistics Estonia
Finland 2018	112		882 867	5 517 919	12.69	2.03	This study
Sweden 2018		150‐180 in SNPA	1 761 190	10 040 995	8.51‐10.22	1.49‐1.79	Tedroff K., personal communication
Finland 2018		195 in FCNA	882,867	5,517,919	22.1	3.53	This study

Note

FCNA, Finnish Pediatric Neurological Association; SNPA, Swedish Neuropediatric Association.

^a^ Population data from 2011 census.

The prospects for more child neurologists in the United States seemed ominous in 2005^11^ and appear to have been realized^12^. In contrast, a certain oversupply was reported from Canada.^13^ In Finland, despite a substantially higher density of child neurologists, no risk of oversupply is foreseeable. The present study showed a wide variation in the geographic distribution of child neurologists. Typical of metropolitan areas^3^, there was a relatively high concentration of both hospital‐based and private office‐based specialists in metropolitan Helsinki.

Why child neurology as a specialty is so popular in Finland may be explained by several reasons: first, a long habilitation tradition of children with long‐term diseases in general and neurological diseases in particular. Thanks to a distinguished and very influential professor of pediatrics at the University of Helsinki, Arvo Ylppö, three hospitals (“children's castles”), including the Children's Castle Hospital in Helsinki, were erected since 1920 in Finland. Those hospitals worked parallel to the National Health Service system Their work was further supported by the National Association for Habilitation of Neurologically Handicapped Children and its countrywide network of regional boards with child neurological expertise. Today, their activities are fused to the National Health Service system. Second, many rare hereditary diseases, including neurological ones, occur in the Finnish population in a relatively larger proportion than in other populations (“the Finnish diseases heritage”).^14^ This fact is likely to have catalyzed interest in clinical child neurology and challenged methods of neurometabolic diagnostics. Third, there were some few active neurologists, who saw the contemporary shortenings and needs for development of child neurology as part of general pediatric specialist education. Fourth, one further reason might be the very well‐organized clinical work and different kinds of educational opportunities. Finally, the position of child neurology among the main specialties was already in 1974 written in the commission report on the future of clinical neurosciences in Finland.

A half of the cases referred to tertiary inpatient care come from health centers. In the study of Haataja and Sillanpää^15^, the referring sources to the Turku University Hospital during 1993‐1998 were health center in 48%, from inside the hospital, virtually all from the department of pediatrics (20%), CN private clinic (9%), municipal CN specialist clinic (8%), other hospital (8%), and unknown (7%). The referring doctor was a health center general practitioner in 47%, pediatric generalist in 25%, child neurologist in 8%, and someone else in 22% of cases (including 0.5% of adult neurologists). The main diagnosis at hospital discharge was developmental disorder in 43%, migraine or other headache in 18%, epilepsy or febrile seizures in 14%, behavioral disorder in 10%, and other diagnosis in the remaining 15%.^15^

As to referrals of children with seizures, it has long been a rule in Finland that children with any seizures to be admitted to an appropriate hospital outpatient clinic for evaluation.^16^, ^17^ In case of a first complicated febrile or nonfebrile epileptic seizure, the vast majority of the children come to a hospital emergency unit without any preceding medical contact. According to the recent Finnish practice recommendations from the year 2013 and again 2020^18^, children with any first nonfebrile or complicated febrile epileptic seizure are invariably admitted to hospital, and children with simple febrile seizures preferably treated as outpatients by general practitioners or experienced pediatricians.

As one of the recognized full specialties in Finland, child neurology has a firm status in the Finnish healthcare system. Two years of general pediatrics in the training program enables to maintain academic and clinical ties with general pediatrics. Recruitment of trainees to fill the vacancies in the future also looks favorable so far. Importantly, CN services, even though unevenly distributed in the country in terms of specialists per population, are obviously well accessible as measures with overall short waiting time and reimbursed communications in sparsely populated areas.

In Finland, pediatricians are traditionally neither interested nor trained in treating epilepsy that is one of the cornerstones of child neurology. According to a Finnish population study, 44% of all children with epilepsy or any long‐term disorder are in need of neurological consultation.^19^ Children and adolescents with epileptic seizures are treated by child neurology specialists. In the absence of trained pediatric habilitation doctors, the treatment and habilitation fall to child neurologists. In the Finnish system, child neurologists also follow‐up children at risk of developmental delay or disorder. The important threefold role of child neurologists as “acutologist,” “habilitationist,” and “developmentalist,” added with the very close daily collaboration with pediatricians, is likely to do that the full specialty of child neurology is not questioned in Finland. All the training positions are continuously occupied. The needs of child neurologists and child neurological services seem to be rising, not declining.

There are, however, some challenges in the field of child neurology. The rapid expansion of CN training in the 1980s and 1990s makes that many child neurologists will retire within a short time period, and the demands for substituting specialists will be unusually high. More recruitment and training are necessary. The current CN education is faced with both quantitative and qualitative challenges. A greater emphasis in teaching is to be put on developmental and autism spectrum disorders, fetal and neonatal neurology, genetics, and neuroimmunology. A prerequisite for filling the demands is sufficient opportunities for baseline and continuous specialist training. Teachers in the CN education programs, while expected to be working as clinicians, must also be offered opportunities for research.

To conclude, the modern Finnish child neurology has a more than fifty‐year history. It has been a recognized full specialty for 40 years. The six‐year training program is in harmony with that of the European Union countries. The child neurologist density clearly exceeds the international recommendations. Children with onset of epilepsy are invariably primarily treated, whether outpatients or inpatients, by a trained child neurologist. Although some variation exists, the countrywide coverage of child neurological services is very satisfactory fulfilling the international recommendations in even sparsely populated areas.

## CONFLICT OF INTEREST

None of the authors has any conflict of interest to disclose. We confirm that we have read the Journal's position on issues involved in ethical publication and affirm that this report is consistent with those guidelines.
